# Neural classification maps for distinct word combinations in Broca’s area

**DOI:** 10.3389/fnhum.2022.930849

**Published:** 2022-11-03

**Authors:** Marianne Schell, Angela D. Friederici, Emiliano Zaccarella

**Affiliations:** ^1^Department of Neuropsychology, Max Planck Institute for Human Cognitive and Brain Sciences, Leipzig, Germany; ^2^Department of Neuroradiology, Heidelberg University Hospital, Heidelberg, Germany

**Keywords:** Broca’s area, language, phrasal processing, fMRI, MVPA

## Abstract

Humans are equipped with the remarkable ability to comprehend an infinite number of utterances. Relations between grammatical categories restrict the way words combine into phrases and sentences. How the brain recognizes different word combinations remains largely unknown, although this is a necessary condition for combinatorial unboundedness in language. Here, we used functional magnetic resonance imaging and multivariate pattern analysis to explore whether distinct neural populations of a known language network hub—Broca’s area—are specialized for recognizing distinct simple word combinations. The phrases consisted of a noun (flag) occurring either with a content word, an adjective (green flag), or with a function word, a determiner (that flag). The key result is that the distribution of neural populations classifying word combination in Broca’s area seems sensitive to neuroanatomical subdivisions within this area, irrespective of task. The information patterns for adjective + noun were localized in its anterior part (BA45) whereas those for determiner + noun were localized in its posterior part (BA44). Our findings provide preliminary answers to the fundamental question of how lexical and grammatical category information interact during simple word combination, with the observation that Broca’s area is sensitive to the recognition of categorical relationships during combinatory processing, based on different demands placed on syntactic and semantic information. This supports the hypothesis that the combinatorial power of language consists of some neural computation capturing phrasal differences when processing linguistic input.

## Introduction

When processing connected speech, our linguistic combinatorial capacity must be flexible enough to make sense of infinite word combinations (Ding et al., 2016; Sheng et al., 2019). Linguistic combination, however, is not a process where each word can combine with another at random; on the contrary, they are bound by systematic relationships between grammatical categories (Rizzi, 2012). A basic hypothesis is that the language combinatorial faculty contains a neural computation that is sensitive to these relationships distinguishing different phrases. Understanding how different phrases are processed during linguistic combination in the mature brain complements studies on the development of the combinatorial productivity in children (Yang, 2013), and the potential gap between human and non-human linguistic combinatorial capacities (Girard-Buttoz et al., 2022).

Word combination refers to any kind of compositional process between words through which linguistic expressions are organized (Heim, 1982; Heim and Kratzer, 1998; Partee, 2010). Studies on the brain bases of word combination have traditionally focused on the comparison between sentences (or phrases) and pseudo-sentences vs. unstructured word-lists used as non-combinatorial controls (Mazoyer et al., 1993; Stowe et al., 1999; Friederici et al., 2000; Humphries et al., 2005, 2006; Jobard et al., 2007; Zaccarella and Friederici, 2015; Matchin et al., 2017; Zaccarella et al., 2017a). Meta-analytical examinations across a variety of different studies revealed that word combinations in sentences and phrases involves a large fronto-temporal-parietal network, including Broca’s area—pars opercularis (Brodmann Area, BA44) and pars triangularis (BA45)—in the inferior frontal gyrus (IFG) and the inferior part of the posterior superior temporal sulcus/gyrus (pSTS/STG, BA22), as major hubs, and in addition the angular gyrus (AG, BA39); the middle/inferior temporal gyrus (MTG, BA21/BA22) and the temporal pole (TP, BA38; Zaccarella et al., 2017b) depending on task and stimulus material. Within this network, the different regions appear to contribute to combinatorial processing depending on the nature of the linguistic information carried by the words forming the sequence. Neural assemblies in the MTG and in the AG have been shown to be sensitive to the amount of semantic information contained in phrasal and sentential stimulus material—thus being specifically active during compositional processing involving real word semantics compared to pseudowords (Pallier et al., 2011; Graessner et al., 2021b). Broca’s area and the pSTG/STS appear to be conversely involved in syntactic processing independently of semantic information for both real sentences and jabberwocky sentences in which content elements are replaced by pseudowords leaving the functional elements (Friederici et al., 2000; Pallier et al., 2011; Goucha and Friederici, 2015; Matchin et al., 2017).

A closer look at Broca’s area at the lowest level of linguistic complexity seems to show that single word processing and simple composition involves to a certain degree BA44 for syntactic processing (Zaccarella and Friederici, 2015) and BA45 for semantic processing (Graessner et al., 2021b). The involvement of BA45 was reported for simple two-word combinations with increasing semantic load, when comparing meaningful (*fresh apple*) or anomalous (*awake apple*) phrases with syntactically legal pseudo-phrases (*fresh gufel*; Graessner et al., 2021b). Conversely, when syntactic pseudo-phrases (*this flirk*) were contrasted with sequences perceived as lists lacking syntactic information (*apple flirk*), BA44 was conversely found (Zaccarella and Friederici, 2015). In light of these considerations, the major advantage of using simple two-word combination lies in the possibility to focus on the fundamental question of how lexical and grammatical information contribute to the combinatory operation of phrase building at the very basic level in Broca’s area, and to evaluate whether the selective involvement of BA44 and BA45 reflects the processing of distinct lexical categories forming the phrase. Electrophysiological and hemodynamic studies on simple linguistic processing have begun to provide initial evidence that distinct types of word combinations may be differently represented on the cortex (Del Prato and Pylkkanen, 2014), and that within Broca’s area, BA44 and BA45 are differentially active depending on the amount of semantic and syntactic information involved in phrase building (Schell et al., 2017). An independent line of research has further shown that the strength of cortical entrainment tracking phrasal presentation rate during auditory language processing is sensitive to grammatical category information (Burroughs et al., 2021), thus suggesting that the human combinatorial capacity treats syntactic categories differently when processing linguistic input. This functional dissociation mirrors theoretical considerations indicating that words of different grammatical categories play distinct roles in the sentence (Baker, 2003; Rizzi, 2012). Content class categories—nouns, verbs, adjectives, and adverbs—primarily carry descriptive content by denoting entities and events expressed in the sentence. On the contrary, function words—determiners, conjunctions, prepositions, pronouns, and auxiliaries—principally provide grammatical features and purely formal relational properties, thereby creating the syntactic skeleton for grammatically correct sentences. Content and function words thus place different demands on syntactic and semantic processing in language. Empirically, early psycholinguistic investigations already suggested different underlying processing systems for content and function words, reflected in lower levels of accuracy during the recognition of function words—irrespective of frequency—in the adult healthy populations (Bradley and Garrett, 1983) and in agrammatic patients (Friederici, 1985; Shapiro and Jensen, 1986). This differential behavioral pattern is supported by electrophysiological findings showing that the processing of the two vocabulary types correspond to different activity distributions across the cortex (Pulvermuller et al., 1995).

A strong association between executive semantic control and left aIFG has been proposed (Whitney et al., 2011; Noonan et al., 2013; Ralph et al., 2017; Chiou et al., 2018). Executive semantic control has been tested using various tasks—e.g., by asking participants to retrieve semantic information precisely, or by monitoring and selecting semantic information among alternatives (Binder and Desai, 2011; Whitney et al., 2012; Noonan et al., 2013). Similarly, the aIFG is significantly recruited during explicit semantic judgments when the plausibility of real word vs. pseudoword stimuli is evaluated (Graessner et al., 2021a). The anterior part of Broca’s area (BA45) in the IFG, has been found to be modulated by semantic information when, for example, subjects are asked to evaluate the propositional meaning of the sentence (Zhu et al., 2009; Newman et al., 2010; Zaccarella et al., 2017a), or have to solve semantic ambiguities by selecting the appropriate word meanings within the sentence (Rodd et al., 2005; Vitello et al., 2014). In this respect, one task that it is often used to probe explicit semantic control beyond sentential level is the synonym judgment task, where pairs of words are put into relation according to shared/overlapping conceptual-semantic features. Synonym judgment has been widely used in both healthy and clinical populations (Jefferies et al., 2009; Mollo et al., 2016) and has actually been found to be affected in patients presenting semantic aphasia with impaired semantic control, following lesions extending to the left inferior-frontal cortex (Thompson et al., 2018). One advantage of using the synonym judgment task is the possibility look at semantic control below the propositional sentential level and to work with real linguistic stimuli, where the similarity between pairs of synonymous words can be robustly assessed with bidirectional ratings (Whitten et al., 1979). This avoids the generation of pseudoword/non-word lists that although automatically normed (Keuleers and Brysbaert, 2010), never fully exclude possible orthographic, phonological or semantic associations with neighboring real words when inspecting semantic processing.

Syntactic aspects of language processing appear to conversely involve neural populations in BA44 in some studies (Friederici et al., 2006; Makuuchi et al., 2009; Goucha et al., 2017; Wu et al., 2019), or in BA45 in some other (Hagoort, 2005; Tyler and Marslen-Wilson, 2008; Pallier et al., 2011; Matchin et al., 2017; Matchin and Hickok, 2019). Reviews on several imaging studies have proposed a functional association between syntax-based evaluation tasks and the posterior IFG, in particular BA44 (Heim, 2008; Friederici, 2011), e.g., when participants assess morphosyntactic information—like syntactic gender agreement between determiner and noun, as in der Baum [correct: *the*(masc) *tree*(masc)] vs. das Baum [incorrect: the(neutrum) tree(masc); Hammer et al., 2007; Heim et al., 2010], phrasal status (Zaccarella et al., 2017a), or word-class information—like in category violation contexts, as in er kniet [correct: *he kneels*(verb)] vs. er Knee [incorrect: *he knee*(noun); Herrmann et al., 2012]. A task that has been extensively used to test aspects of syntactic processing is the word-class judgment task, where words are allocated to a certain class according to specific categorical features or syntactic roles defining it (e.g., tense, aspect, agenthood, etc.). Word-class judgment task is for example used when testing the impact of word-class prime perception on target words (Berkovitch and Dehaene, 2019; Pyatigorskaya et al., 2021). These latter studies on the subliminal or supraliminal perception of certain word-class primes suggest that participants are able to retrieve abstract linguistic information that splits words into syntactic classes according to their role in the sentence and that is used to construct grammatical sequences according to the syntactic rules of the language in question. In this sense, an advantage of the explicit word class judgment task is that it decreases the automaticity of syntactic processing, which is known to be highly automatic in adults (Hahne and Friederici, 1999), to observe the neural populations involved during attention to the categorical labels (Det, N, V, etc.) that need to be accessed to successfully combine words.

The goal of the present study is to directly link neural responses in Broca’s area to basic phrasal combinations using simple two-word combinations in a functional magnetic resonance imaging (fMRI) setting applying a multivariate pattern analysis as decoding method. Mimicking previous experimental designs on basic word composition (Del Prato and Pylkkanen, 2014; Schell et al., 2017) two phrasal types of equal syllabic length are used in the present experiment. A single noun (flag) could either form a noun phrase (NP) with an attributive adjective (green flag) or form a determiner phrase (DP) with a determiner (that flag). Note that here we use the DP notation to indicate the determiner + noun phrase, to illustrate the distinction between the two types of phrases investigated here—for the interested reader, an introduction to the DP- vs. NP-hypothesis for determiner + phrase constructions is provided in Bernstein (2008). We follow influential linguistic theories suggesting that articles (e.g., the), demonstratives (e.g., this), quantifiers (e.g., some) and other numerals (e.g., two) belong to the DP system (Zamparelli, 2014). The purpose of this work is to focus on the similarity of function across determiners, to investigate how the combinations between distinct categories (here determiners and adjectives with nouns) may be differentiated at the neural level (see section “Stimuli list” in Supplementary material).

Using a voxel-based classifier to capture the relationship between the spatial pattern of fMRI activity and experimental conditions, we searched for areas containing detailed information of category-specific phrasal processing within Broca’s area neural populations based on prior work suggesting this area as crucial for phrase processing. Such analysis is affine to theories of neural representation for population codes, where information is encoded in patterns of activity across large neuronal populations (Georgopoulos et al., 1986; Pouget et al., 2000). In the present case, we sought to understand how each voxel, within the independently defined anatomical maps for BA44 and BA45, directly contributed to the classifiers. More specifically, which voxels contributed to classifying the phrases included in our DP sample as DP phrases and which voxels contributed to classifying the phrases included in our NP sample as NP phrases. We did this within progressively larger zones of prediction accuracy. We reasoned that, if Broca’s area is sensitive to phrasal processing, it should first be able to classify phrasal combinations significantly above chance compared to a corresponding control region. Second, if the region is sensitive to phrasal differences, distinct neuronal populations inside the area should be recruited. In this case, spatial localization should adhere to cytoarchitectonic subdivisions proposed for the region (Amunts et al., 1999). Here, we specifically expected neuronal populations associated with adjective + noun combinations to be localized in BA45. Neuronal populations associated with determiner + noun combinations were expected to be localized in BA44. Third, we wondered whether this functional dissociation could be independent of task manipulations, which are also known to involve the left inferior frontal regions and have been discussed previously, also in the wake of previous evidence demonstrating effects of task demands on the same stimulus sets (Caplan, 2010). We therefore asked our participants to perform two types of tasks while listening to DP and NP phrases in the scanner, which we presented as pairs of two-word spoken phrases where the noun of the second phrase could be a repetition, a synonym, or an unrelated noun to the noun of the first phrase. For half of the experiment, the participants performed a synonym judgment task on the trials—we refer to this task as the *semantic task (SEM)*, for the other half they performed a word-class judgment task—we refer to this task as the *syntactic task (SYN)*. Overall, evidence in favor of a functional subdivision in Broca’s area would support the claim that Broca’s area is associated with linguistic combination and that phrasal specificities might lie in the neuronal populations of BA44 and BA45 (Zaccarella et al., 2017b).

## Materials and methods

### Participants

Twenty-eight native German speakers (13 females and 15 males; mean age: 28.25 years, standard deviation (SD) 4.03) were recruited from an internal database and invited to participate twice in the study. All participants were right handed as assessed with the Edinburgh questionnaire (Oldfield, 1971), reported normal hearing and had normal or corrected-to-normal vision. None of them had any history of neurological or psychiatric disorder. The study was approved by the Research Ethics Committee of the University of Leipzig and followed the guidelines of the Helsinki declaration. Written informed consent was obtained from all participants according to the procedures approved by the Research Ethics Committee of the University of Leipzig. All participants received monetary compensation after completing the experiment.

### Experimental design

Our functional study manipulated two factors in a 2 × 2 factorial design (see Table 1). The first factor was PHRASE (NP: adjective + noun = “blue boot”; DP: determiner + noun = “this boot”). The second factor was TASK (SEM: “Word meaning” for the semantic task; SYN: “Word class” for the syntactic task).

**TABLE 1:** Experimental design.

		Phrase			
		Det + noun (DP)		Adj + noun (NP)	
**Task**	SYN	Diese Fahne *This flag*	Jene Fahne *That flag*	Lange Fahne *Long flag*	Grüne Fahne *Green flag*
	SEM	Diese Fahne *This flag*	Jene Fahne *That flag*	Lange Fahne *Long flag*	Grüne Fahne *Green flag*

The study crossed type of PHRASE and type of TASK in a 2 × 2 factorial design. The first factor was PHRASE (DP: determiner + noun = “this flag”; NP: adjective + noun = “green flag”), the second factor was TASK (SYN: “Word class” for the syntactic task; SEM: “Word meaning” for the semantic task).

### Stimuli

The experiment was carried out in German. Each participant received a separate list of items from a set of possible DP or NP combinations generated by combining determiners and adjectives with all pairs of synonyms that we had obtained from bidirectional ratings in an independent group of 18 healthy participants (9 female, age: mean = 24.7, SD = 2.8). To test bidirectional ratings, we selected all bi-syllabic nouns from the Leipziger Wortschatz database^1^ and removed abstract concepts or nouns having any intrinsic color that could not easily be matched with color adjectives (e.g., animals or fruits). We then selected 106 pairs of plausible synonyms. All synonyms were matched with an unrelated word according to gender. All synonym and unrelated pairs were shown in randomized order on a computer screen (the first noun for 2 s, the second noun until button press) in both forward order and reverse order. Once the second noun appeared on the screen, participants were asked to judge the semantic similarity of the pair on a 7-point Likert scale, starting from 1 “not at all similar” to 7, “very much similar.” We selected 12 unique pairs of synonyms with the highest ratings of semantic similarity (>6) and no significant effect for directionality for the functional MRI experiment. Additionally, we chose the corresponding unrelated word with the lowest similarity rating (<2; see section “Bidirectional rating” in Supplementary material). The final pool of items was matched for syllabic length and controlled for bigram frequency using the google web1t database containing n-gram counts for approximately 100 billion word-tokens for the German language (Linguistic Data Consortium, University of Pennsylvania). Average bigram frequencies (log) across participants were 2.28 (SD: 0.10) for the DPs and 2.12 (SD: 0.10) for the NPs, with an averaged effect size (*r*) of 0.018 (CI = −0.14 to 0.17; see Supplementary Figure 1; Sassenhagen and Alday, 2016). For the DP condition we had simple determiners followed by nouns with relative inflectional gender marking: dies + er (masculine)/ + e (feminine)/ + es (neutrum), “this”; jen -er/-e/-es, “that”; jed -er/-e/-es “each”; manch -er/-e/-es, “some”. For the NP condition we had simple qualifying adjectives for the first phrase (lang -er/-e/-es, kurz -er/-e/-es, groß -er/-e/-es, klein -er/-e/-es, English: long, short, big, small with their respective inflectional ending) and color adjectives for the second phrase (rot -er/-e/-es, gelb -er/-e/-es, blau -er/-e/-es, grün -er/-e/-es, English: red, yellow, blue, green), counterbalanced across grammatical gender (see section “Stimuli list” in Supplementary material).

### Procedure

A trial always consisted of pairs of spoken two-word phrases (see section “Audio recordings” in Supplementary material). The noun of the second phrase was either a repetition, a synonym, or an unrelated noun of the noun in the first phrase (see section “Stimuli list” in Supplementary material). Each experimental run started with an instruction screen (“Word meaning” or “Word class”) and consisted of a sequence of 12 trials. Each run always contained three DP trials, three NP trials, three mixed trials, and three filler trials in randomized order. Answers were recorded by pressing the button box placed inside the scanner at the end of the second phrase. For each run, there were always 6 “yes” responses and 6 “no” responses. Importantly, DP trials always contained two phrases both formed by determiners and nouns, NP trials always contained two phrases that were both formed by adjectives and nouns, while mixed trials contained either a DP followed by an NP, or the other way round. In the SYN, participants judged whether the second phrase consisted of the same word class elements as the ones contained in the first phrase of the trial. For example, the DP trial JENE FAHNE | DIESE FLAGGE would receive an affirmative answer, while the mixed trial JENE FAHNE | BLAUE FLAGGE would receive a negative answer, since there is a noun in one case and a determiner in the second case. The phrases were thus compared according to specific categorical features or syntactic roles that define the word classes to which the internal words belong. In the SEM, participants were asked to judge whether the second phrase could refer to the same object described by the first phrase of the trial. For example, the DP trial JENE FAHNE | DIESE FLAGGE would receive an affirmative answer since they both refer to the same object (*flag*), while the mixed trial JENE FAHNE | BLAUE DOSE would receive a negative answer, since they refer to different objects (*flag* and *can*, respectively). The phrases, in this case, were thus compared based on possible overlapping conceptual-semantic features between them. Given the nature of the planned fMRI analysis, the answers for the DP and the NP trials during the SYN task were always “yes” since both trials had to contain the exact same word classes in order to be coded as trials of interest. In contrast, the answers for the DP and NP trials during the SEM task were “yes” answers two thirds of the time. This because a trial with same word classes could be coded as trial of interest, even when they would not refer to the same object. In order to fully counterbalance the “yes/no” occurrences across tasks and runs, we inserted filler trials like JENE FAHNE | DIESER JENER to counterbalance “no” answers for DP trials and mixed trials BLAUE FAHNE | DIESE FLAGGE to counterbalance “yes” answers for the NP trials. Overall, upon hearing the first phrase of a trial, participants had a 50% change to press “yes” and a 50% change to press “no” across type of phrase and task. The presentation of each trial was time-locked with volume acquisition, which is indicated as beneficial for multivariate analyses (Etzel et al., 2009) and lasted on average 3,400 ms. We split the whole experiment into two experimental sessions on two separate days lasting 28 min each. Each session contained 144 trials in twelve alternating task blocks. On each day participants performed 40 trials of each task outside the scanner to get familiar with the experimental setup. Training stimuli were not used inside the scanner. The experiment was implemented in Presentation software package (Neurobehavioral Systems, Inc., Berkeley, CA, USA^2^).

### Data acquisition

MRI data were obtained using a 3-Tesla Siemens Magnetom Prisma scanner (Siemens Medical Systems, Erlangen, Germany) with a 20-channel head coil at the Max Planck Institute for Human Cognitive and Brain Science in Leipzig, Germany. To reduce fatigue the functional images were acquired on two separate days, in total, 1,554 T2*-weighted echo-planar images (777 images for each scan day, TR = 2,110 ms, TE = 22 ms). Each volume consisted of 40 axial slices, parallel to AC-PC line, in ascending direction, and with whole brain coverage. Scans had an in-plane resolution of 3 × 3 mm and a 2.5 mm slice thickness with an inter-slice gap of 0.5 mm (flip angle 90°, field of view 192 mm, matrix size 64 × 64).

### Behavioral data analysis

Performance was assessed by calculating accuracy and reaction times across trials using the response of the first scanning day, since the button presses could not be registered during the second scanning day for some subjects due to a technical error in the behavioral recording system. These participants had performed at ceiling during the first scanning day and self-reported to have accomplished the task without any difficulty on the second scanning day. Participants with an overall accuracy below 80% (*N* = 5) were excluded from the further analysis. One additional participant was excluded due to excessive movement in the scanner (see below). Twenty-two participants (11 females and 11 males; mean age of 27.86 years, SD of 3.99) were included in the analysis of the imaging data. Statistical analyses were performed in R (version 3.6.1) with the generalized linear mixed-effects model with fixed effect for condition and task and random factor for participants, using the lme4 package (Bates et al., 2015). We computed a mixed logit regression for the error data analysis. Statistical significance was tested using the “multcomp” package (Hothorn et al., 2008).

### Imaging data preprocessing

Preprocessing was conducted with SPM8 (Wellcome Imaging Department, University Collage, London, UK^3^) as implemented in MATLAB 7.14.0.739 (R2012a, Mathworks, Inc., Sherborn, MA, USA^4^). For each day, functional images were realigned to the first image and unwrapped with an accompanying fieldmap and corrected for movement induced variance by rigid and non-rigid transformation. Subsequently, the functional images were slice time corrected with the middle slice as a reference, co-registered to participants’ individual T1-weighted image (TI = 650 ms; TR = 1,300 ms; alpha = 10°; FOV = 256 × 240 mm), acquired in a previous session, and normalized to MNI space using unified segmentation with a resampling of the images into 3 mm isotropic voxels. The five initial volumes were excluded to allow for magnetic saturation effects. The unsmoothed images were handed to the decoding toolbox for multivariate analysis to preserve fine-grained subject-specific information pattern (Kamitani and Sawahata, 2010).

### First level analysis

In order to investigate the neural encoding of the trial types, a General Linear Model (GLM) was estimated using three runs of normalized data for each day, resulting in six runs for the whole experiment. Each run comprised four task blocks, two of each task, with six trials per condition, so that the experiment was fully balanced between the runs. A regressor for each condition and each run was entered. All regressors were locked to the onsets of the trial, while the duration were set to cover the full length. We added the movement parameters as separate regressors of no interest to the model. The resulting 24 beta images (6 runs × 4 conditions) were used for the multivariate pattern analysis.

### Multivariate pattern analysis

We used “The Decoding Toolbox,” TDT (Hebart et al., 2014) to determine the decision boundary for the different conditions within the data. The toolbox implements LIBSVM software (Chang and Lin, 2011) to train a linear support vector machine (SVM) with a decision function of a fixed cost parameter, c = 1. We applied two different classification approaches, an ROI method to evaluate Broca’s area and a searchlight method for whole-brain classification. Both approaches used a stratified, leave-one-run-out, cross-validation (sixfold by 6 times classification) always using 5 runs for the training set (20 beta images, 10 for each class) and the “left-out” sixth run to evaluate the data (4 beta images, 2 for each class) and containing novel items of the same conditions. This is repeated several times as a function of the numbers or runs included in the study and averaged afterward to obtain a mean decoding value of informativity, that allows inferences about the representational content of a certain region (Haynes and Rees, 2006). The first classification was done alone with the PHRASE factor to see which patterns were able to classify participants as listening to DPs or to NPs. We assigned all DPs—irrespective to task—to class *+*1 and all NPs—again, irrespective to task—to class −1. Here, class number refers to the direction of the binary decision during the classification processing. The second classification was done along the TASK factor. Here, all trials of the SYN were assigned to class *+*1 and all trials for the SEM were assigned to class −1. Threshold for significance was set at p < 0.05.

### Broca’s area decoding analysis

Broca’s area (see Supplementary Figure 2) was composed using the left area 44 (BA44) and area 45 (BA45; Amunts et al., 1999) from the SPM Anatomy toolbox v 1.8 (Eickhoff et al., 2005) and assessed *via* the GUI. We refer specifically to the anatomical mask as IFG in the following statistical analyses. The left primary visual cortex (V1, BA17) from the same toolbox was used as control region to validate the classification approach (Etzel et al., 2009). The images were co-registered so that the number of voxels used for the classification analysis was similar across the participants (IFG with 421 voxels, V1 with 400 voxels). For each participant and classification, we extracted: one accuracy value per ROI and several SVM pattern values, one for each voxel within the ROI (see below). Both were determined by averaging the values across the folds.

### Broca’s area classification accuracy

For both Broca’s area and V1 we calculated the classification accuracy. A one sampled *t*-test was used to calculate the significance of the classification against the chance level of 50%. A second paired *t*-test was performed to test whether the classification in Broca’s area performed better than in V1. We additionally analyzed the mean specificity for each class separately to assess any asymmetry in classifier behavior *via* two-sided paired *t*-tests.

### Broca’s area classification maps

While the accuracy refers to the success rate of the classification, SVM pattern determines the contribution of a voxel to the final classifier function—also implemented in TDT toolbox—to get the positive and negative distance of the separating hyperplane (Haufe et al., 2014). High positive values for a voxel corresponded to high contribution to classify a stimulus as being a DP (or SYN TASK). Similarly, high negative values for a voxel corresponded to high contribution to classify a stimulus as being an NP (or SEM TASK). This method has been already successfully tested on different kinds of linguistic processes, as for example for understanding how each voxel in the anterior/superior temporal lobe directly contributed to the classifiers’ prediction in favor of intelligible vs. unintelligible trials during natural and rotated speech perception (Evans et al., 2014). All SVM pattern values were averaged across participants, resulting in one value per voxel. As the voxels with the largest magnitude contribute the most to the classification results, we first selected the 5% (21 voxels), 10% (42 voxels) and 15% (63 voxels) of largest positive and negative values for both classification analyses (Evans et al., 2014). We then obtained the classification maps by localizing the same voxels within BA44 and BA45 to assess the distribution of the SVM pattern for the different conditions in Broca’s area. Statistical significance of the observed voxel distribution was tested with a χ^2^-test. A Pearson correlation coefficient between condition and class classification, was also computed on the corresponding SVM patterns obtained from the maps described above to obtain a measure of Broca’s area correlational specificity.

### Whole-brain searchlight analysis for PHRASE and TASK classifications

Whole-brain analyses were performed to create overall information maps to show the pattern differences across the brain for both PHRASE and TASK classification. Thereby, accuracy values were assigned to the central voxel within each spherical searchlight of 12 mm (equals 4 voxels), iteratively performed over all voxels in the brain, resulting in a 3-dimensional accuracy map. Group analysis was performed by combining the individual subject maps with a *t*-test at each voxel as implemented in SPM8 to evaluate the classification approach.

## Results

### Behavioral data results

Average accuracy rate for the DP condition during syntactic task (DP-SYN) was 0.89 (SD: 0.12) and it was 0.92 (SD: 0.1) during the semantic task (DP-SEM). The average accuracy rate for the NP condition during the syntactic task (NP-SYN) was 0.92 (SD: 0.1) and it was 0.95 (SD: 0.09) during the semantic task (NP-SEM). The logit regression analysis on the accuracy data with factors TASK and PHRASE as fixed effects and by-participant slope for task [acc – task × phrase + (1 + task | subj)] revealed moderates effects for TASK and PHRASE, but no interaction: TASK coefficient_ß_ = −0.5, SE_ß_ = 0.21, *z* = 2.37, *p* = 0.02; PHRASE coefficient_ß_ = −0.21, SE_ß_ = 0.09, *z* = 2.18, *p* = 0.03; TASK × PHRASE coefficient_ß_ = 0.001, SE_ß_ = 0.1, *z* = 0.008, *p* = 0.99. Sum-to-zero contrasts were used for the factors TASK and PHRASE. More complex random structure resulted in overfitted models. The average reaction times of the DP condition during syntactic task (DP-SYN) was 976.3 ms (SD: 476 ms) while it was 872.8 ms (SD: 422.8 ms) during the semantic task (DP-SEM). The average reaction times for the NP condition during the syntactic task (NP-SYN) was 963.1 ms (SD: 470.01 ms) and 849.5 ms (SD: 418.4 ms) during the semantic task (NP-SEM). We fitted a linear mixed effects model with the logarithmic transformation of RT as the dependent variable, with TASK and PHRASE as fixed effects and by-participant slope for task [RT – task × phrase + (1 + task | subj)]. More complex random structure resulted in overfitted models. We found no main effects and no interaction: TASK coefficient_ß_ = 0.04, SE_ß_ = 0.02, *z* = 1.78, *p* = 0.21; PHRASE coefficient_ß_ = 0.01, SE_ß_ = 0.01, *z* = 0.75, *p* = 0.9; TASK × PHRASE coefficient_ß_ = 0.01, SE = 0.01, *z* = 0.56, *p* = 0.96. To note, comparison of the slope between the tasks in the reaction time data showed no different learning trends between the SYN and SEM during the experimental manipulation (see Supplementary Figure 3). As a side analysis, we failed to find RT differences between SEM trials with “yes” answers and SEM trials with “no” using a canonical *t*-test comparison between the two sets with *t*_(1,38)_ = 0.04, *p* = 0.96.

### Broca’s area classification accuracy

**FIGURE 1:**
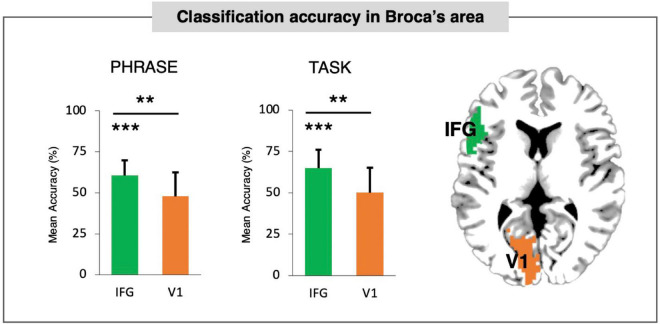
Classification accuracy in Broca’s area. PHRASE classification and TASK classification accuracy for Broca’s area tested against control region (V1/BA17). Both PHRASE and TASK classifications in Broca’s area revealed significant accuracy differences compared to both control region and chance level set at 50%. Bars denote standard deviation (SD). IFG, inferior-frontal gyrus. Bars denote standard deviations. **p < 0.005, ***p < 0.001. Figure created using the Multi-Image Analysis GUI viewer (Mango, Version 4.1; http://ric.uthscsa.edu/mango/) and MATLAB 7.14.0.739 (R2012a, Mathworks, Inc., Sherborn, MA, USA).

We first evaluated the accuracy for Broca’s area (see Supplementary Figure 2) in comparison to control region (V1) for PHRASE and TASK classification. Single overall classification accuracies are reported in Figure 1. Both PHRASE and TASK classifications revealed significant accuracy differences compared to the control region in V1 and chance level set at 50% (PHRASE: IFG against V1: *t*_(21)_ = 3.712; *p* = 0.001; IFG against 50%: *t*_(21)_ = 5.328; *p* < 0.001; TASK: IFG against V1: *t*_(21)_ = 3.239; *p* = 0.004 IFG against 50%: *t*_(21)_ = 4.812; *p* < 0.001). No imbalance in the classifier behavior was found: *p* = 0.141, *t*_(21)_ = 1.530 for the classification between conditions—DP classification of 63.64% (SD = 11.37%), NP classification of 67.58% (SD = 14.74%), and *p* = 0.254, *t*_(21)_ = 1.173 for classification between the tasks—syntactic task accuracy was 67.42% (SD = 15.19%), SEM accuracy was 62.50% (SD = 19.71%).

### Broca’s area classification maps

**FIGURE 2:**
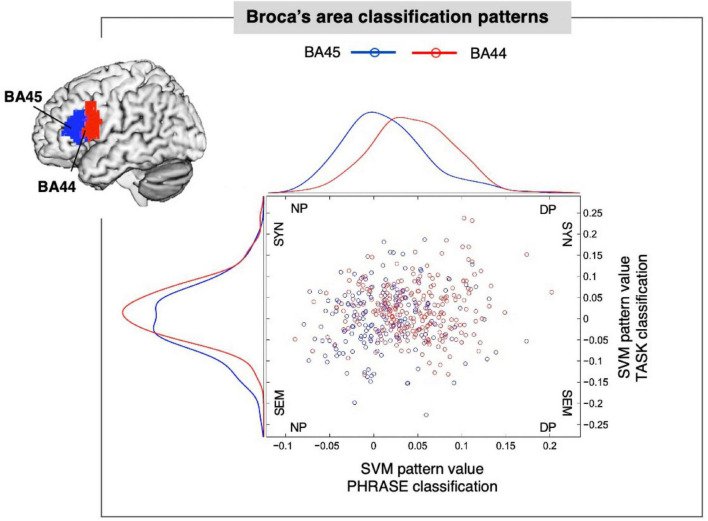
Broca’s area classification patterns. SVM (support vector machine) pattern values within the anatomically defined Brodmann Area (BA) 44 and BA45 of Broca’s area (top-left), expressing positive and negative distances of each voxel from the separating hyperplane for PHRASE and TASK. Blue dots represent voxels located in BA45. Red dots represent voxels located in BA44. Curves represent pattern distribution for PHRASE and TASK in BA44 and BA45, respectively. SYN, syntax; SEM, semantics; NP, noun phrase; DP, determiner phrase. Figure created using the Multi-Image Analysis GUI viewer (Mango, Version 4.1; http://ric.uthscsa.edu/mango/) and MATLAB 7.14.0.739 (R2012a, Mathworks, Inc., Sherborn, MA, USA).

**FIGURE 3:**
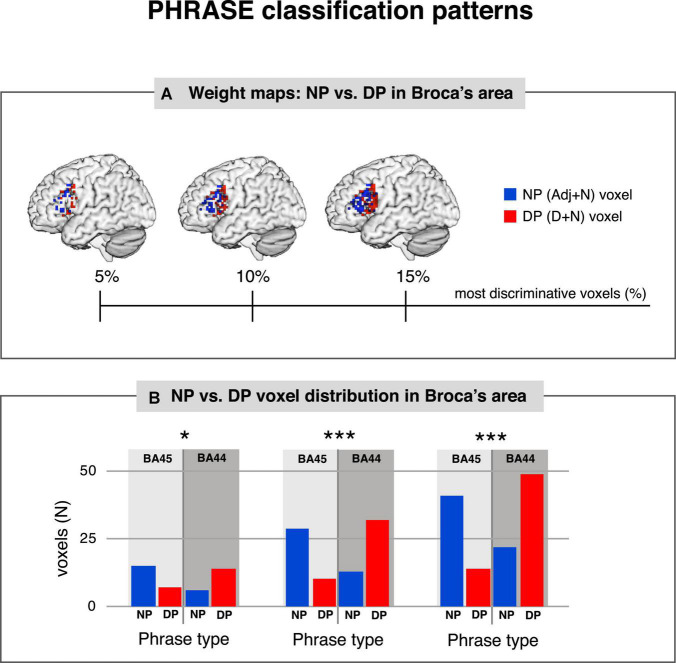
PHRASE classification patterns. **(A)** Weight maps: NP vs. DP in Broca’s area. The most discriminative 5, 10, and 15% of classifier weights from the classifier trained to discriminate DP vs. NP, along the PHRASE factor in Broca’s area. **(B)** NP vs. DP voxel distribution in Broca’s area. PHRASE classification χ^2^-tests showed significantly distinct distributional patterns in Broca’s area for the 5% (left), 10% (middle) and 15% (right) most discriminative voxels, with voxels identifying determiner + noun combinations being strongly located in BA44, while voxels classifying adjective + noun combinations being conversely located in BA45. NP, noun phrase; DP, determiner phrase. *p < 0.05, ***p < 0.001. Figure created using the Multi-Image Analysis GUI viewer (Mango, Version 4.1; http://ric.uthscsa.edu/mango/) and MATLAB 7.14.0.739 (R2012a, Mathworks, Inc., Sherborn, MA, USA).

We then asked whether voxel contribution to each specific class followed the cytoarchitectonic organization of area 44 and 45 within Broca’s area. We used the SVM pattern values (Haufe et al., 2014) to show the contribution of each voxel to the representation of the class under analysis. We chose 5, 10, and 15% of the highest positive and negative values corresponding to the most discriminative voxels for each classification. Finally, we assigned each of the selected voxels back to either BA44 or BA45, to assess the distribution of the SVM pattern for the different conditions (see Figure 2). For the PHRASE classification χ^2^-tests showed significantly distinct distributional patterns in Broca’s area, with voxels identifying determiner + noun combinations being strongly located in BA44. Voxels classifying adjective + noun combinations were conversely located in BA45 (5% χ^2^(1) = 6.11, *p* = 0.013; 10% χ^2^(1) = 17.28, *p* < 0.001; 15% χ^2^(1) = 23.52, *p* < 0.001). Cramèr’s *V* magnitude of relationship test (Rea and Parker, 1992) revealed a relatively large association strength between the type of phrase and anatomical region for three subset distributions (*V*_5%_ = 0.38; *V*_10%_ = 0.45; *V*_15%_ = 0.42; see Figure 3). This indicates that the type of condition had a strong effect on whether the location was in BA44 or BA45. Please note that complete weighting of all voxels within Broca’s area are in Figure 2, while this additional parametric analysis makes use of the most discriminative voxels within the region using the tails of the distribution of the voxels within the region. The 5, 10, and 15% thresholds are arbitrary choices we selected to parameterize the analysis and to localize the most discriminative voxels, within a region containing only a moderate number of overall voxels given the slice thickness involved. For the TASK classification, χ^2^-tests revealed significant distributional patterns for the same regions (5% χ^2^(1) = 7.78, *p* = 0.0053; 10% χ^2^(1) = 8.05, *p* = 0.0045; 15% χ^2^(1) = 8.18, *p* = 0.0042), confirmed by the moderate association strength between type of task and anatomical region (*V*_5%_ = 0.43; *V*_10%_ = 0.30; *V*_15%_ = 0.25; Supplementary Figure 4). We further tested whether there was a linear relationship between PHRASE and TASK classifications to see if the voxels most informative for one classification where also the most informative for the other classification. While the correlation coefficient confirmed a small, positive, but significant relation between the voxels (Pearson: *r* = 0.135, *p* = 0.005), the *R*^2^ of 0.018 suggests that only 1.8% of the variance within the data was explained (see Supplementary Figures 5, 6).

### Whole-brain searchlight analysis for PHRASE and TASK classifications

Whole-brain analyses were finally performed to create overall information maps across the brain for both PHRASE and TASK factors. For the PHRASE classification, the information map on the group level showed a broadly distributed network mainly located in the left hemisphere. This included the superior and middle temporal gyri (MTG/STG), AG, supramarginal gyrus (SMG) and IFG. For the TASK classification the information map showed distributed spatial information patterns in the left and right IFG and in the left AG (see Supplementary Figure 7).

## Discussion

The key finding of this study is that neural populations classifying simple word combinations in Broca’s area, a high-order hub of the linguistic system that governs morphosyntactic relationships among words (Musso et al., 2003; Pallier et al., 2011; Goucha and Friederici, 2015), are not uniformly distributed. Rather, classification involves neuroanatomically distinct sub-regions within the area, reflecting a distinct neural processing for the different phrases included in our experimental sample. Overall, the data appear to be functionally consistent with recent meta-analytical results showing a strong sensitivity of Broca’s region to syntactic processing across languages and structural complexities (Grodzinsky et al., 2021), and offer an attempt to further characterize the region’s involvement in language processing, recapitulating complexity at a finer level of linguistic resolution and neuroanatomical specification.

The present study shows that Broca’s area is sensitive to the recognition of distinct word combinations. Anterior Broca’s area (BA45) contains a significantly larger number of voxels than posterior Broca’s area (BA44) in identifying phrases formed by adjective + noun combinations. Given the rich conceptual content of the adjective + noun classification, we believe that these findings are in agreement with both correlational and causal analyses of semantic load in this area (Hamilton et al., 2009; Hartwigsen et al., 2016; Graessner et al., 2021b). Conversely, the observation that posterior Broca’s area contains a significantly larger number of voxels that are informative for the recognition of determiner + noun combinations with reduced conceptual content, appears to mirror syntactic processing effects found in the area for longer structures, regardless of meaning (Friederici and Kotz, 2003; Bornkessel et al., 2005; Goucha and Friederici, 2015; Zaccarella et al., 2017a; Chen et al., 2021). In this regard, the tasks employed here may not necessarily enhance compositional processing in the phrases we tested. Rather, the results of task comparison irrespective of phrase types might help us to understand the kind of basic linguistic features recognized in anterior and posterior Broca’s regions which are deemed as relevant to the SEM in one case—controlled manipulation/comparison of conceptual-semantic features—and the syntactic task in the other—controlled manipulation/comparison of abstract morphosyntactic features. We believe that future works will have to extend the analysis to the interaction between those tasks particularly enhancing linguistic combination (Pylkkänen, 2019) and the complexity of the phrasal type beyond single word processing in the region (Friederici et al., 2000).

The involvement of Broca’s area for very simple linguistic phrases raises at least two questions concerning both the fact that basic language combinations are largely unaffected in Broca’s aphasics (Grodzinsky, 2000; Grodzinsky and Friederici, 2006), and the compelling evidence that conceptual (adjectival) combination has been associated with a separated linguistic hub involving the anterior temporal lobe (Pylkkänen, 2020). Recent advances in multivariate lesion-behavioral mapping are beginning to provide preliminary answers to both questions, given their more sensitive approach to the association between brain injury and behavioral deficits (Zhang et al., 2014). One of these studies tested simple phrasal comprehension in patients with chronic lesions to the language network after a left hemispheric stroke and diagnosed aphasia (Graessner et al., 2021a). In these patients, both the anterior IFG and the antero-medial temporal gyrus were found to influence phrasal comprehension, however with clear dissociations: lesions to the anterior IFG correlated with reduced semantic decisions, whereas lesions of the antero-medial temporal lobe led to slower decisions with respect to semantic accuracy. This suggests that both regions are likely to contribute actively to linguistic combination in language through complementary roles and that very careful consideration of the methodologies involved and the linguistic nature of the stimulus type is necessary when it comes to simple word combination at the neural level (Maran et al., 2022).

Overall, Broca’s area sensitivity to linguistic sequences is functionally consistent with neural activity measured in the area that correlates with the sound envelop of sentence structures prior to verbalization (Magrassi et al., 2015), or during reading (Nelson et al., 2017). Recently, the investigation of specific time scales for the processing of linguistic units and their neural signatures has shown that the brain is able to track linguistic (phrasal and sentential) dimensions not readily available in the input (Ding et al., 2016; Sheng et al., 2019). The strength of the cortical tracking, however, seems to be modulated by the kind of grammatical category included in the stimulus. This suggests that word-level grammatical information is taken into account to build larger units (Burroughs et al., 2021). In this cited study, in fact, entrainment seems to be significantly reduced when the linguistic stimuli contain phrases formed by different grammatical categories, compared to when the stimuli contain the same grammatical category occurring in a strictly alternating fashion. Much in the same spirit, our work begins to look at the representation patterns of different phrases, complementing previous neurobiological approaches to classify types of linguistic combinations on the cortex (Artoni et al., 2020).

The current findings leave open a few questions. First, here we used a well-controlled, but relatively small sample of determiners and adjectives that were nonetheless combined with a large sample of nouns. This was to reduce inherent baseline issues due to the contrastive nature of the methodology we used (Stark and Squire, 2001), notwithstanding the use of a total number of items similar to other fMRI studies on language employing MVPA techniques (Herrmann et al., 2012). As such, our classifiers were not tested on novel determiners and adjectives that were not included in our stimuli samples, which lessens the generalizability of the present results to more abstract category-based distinction. At the same time, however, the nature of our block design, in which words were never heard in isolation but always as two-word phrases, makes it difficult to draw conclusions about the effects of individual words and category types. Rather, our work provides preliminary evidence that the brain processes incoming phrases differently, depending on the amount of syntactic and semantic information included during the combinatorial process. Past evidence however seems to suggest that a word category effect *per se* may not be enough to explain the neural pattern found for the region in previous studies. A previous work focusing on the comparison between pseudo-DPs (*this flirk*), non-combinatorial DPs made out of determiners and non-pronounceable alphabetic strings (*this xxxxx*) and word-lists of equal syllabic length (*apple flirk*) pointed toward a selective involvement of some subregions of BA44 as the locus of syntactic composition, when the function word effect and the number of words forming the phrase are equally controlled (Zaccarella and Friederici, 2015). An fMRI work related to the previous one and focused on the comparison of DPs against NP phrases and single word controls also showed a clear involvement of subregions of BA44 for DP processing irrespective of phrasal length and stimuli involved (Schell et al., 2017). Some evidence therefore emerges that subregions of Broca’s area may be involved as combinatorial processors within the language system, and that perhaps sections of its posterior part may be specifically involved in the processing of linguistic sequences on the basis of categorical information (Chen et al., 2021). Second, we concur with the idea that linguistic theories make representational hypothesis about the combinatorial mechanisms that the brain needs to implement—including the fact that a certain level of abstraction, the fundamental combinatorial operation works on all categories alike (e.g., Chomsky, 1995; Jackendoff, 2002). However, empirical investigations into natural language in real time suggest that combinatorial processing lead to multiple neural activation in different cortical regions, which cannot always be easily linked back to linguistic theory (Pylkkänen, 2019). This does not mean that certain phrasal constructions bypass syntactic information when not directly relevant—as already claimed in a previous work on adjectival modification and its relationship to the automaticity of syntax (Schell et al., 2017). In the same spirit, the present work has focused specifically on Broca’s area, also for methodological reasons related to the existence of cytoarchitectonic definitions that subdivide the area and the ready availability of anatomical masks that allow for fine-grained analyses within each sub-region of interest. The same is not always true for some of the other anatomical regions of the language system (Amunts et al., 2020). Third, recent advancement in syntactic theory and preliminary behavioral studies, suggest that different phrases may have a much more complex internal structure than previously thought (Zamparelli, 2014; Chesi and Canal, 2019), with different cognitive properties driving internal linguistic differentiations (Agmon et al., 2019). Thus, future investigations should concentrate on systematic, within-category manipulations to explore phrasal and sentential combinations in more detail (Allen et al., 2012; Pylkkänen, 2020; Takashima et al., 2020).

## Conclusion

In our study, we reported functional association between type of word combination and regional information in Broca’s area. We believe that these findings pave the way for a fine-grained characterization of the linguistic features at work during combinatorial processes (Chesi and Canal, 2019), and they generally support spatiotemporal neural models showing that different neural populations might be recruited for different processing demands (Bernacchia et al., 2011).

## Data availability statement

The datasets presented in this article are not readily available because the authors do not have permission to share data according to the ethics agreement. Code is available from the authors upon request. Requests to access the datasets should be directed to MS, marianne.schell@med.uni-heidelberg.de; EZ, zaccarella@cbs.mpg.de.

## Ethics statement

The studies involving human participants were reviewed and approved by the Research Ethics Committee of the University of Leipzig. The patients/participants provided their written informed consent to participate in this study.

## Author contributions

MS, AF, and EZ conceptualized the study and wrote the manuscript. MS collected the data. MS and EZ analyzed the data. All authors discussed results, revised manuscript, and approved it for publication.

## Footnotes

1
https://wortschatz.uni-leipzig.de/

2
https://www.neurobs.com/

3
https://www.fil.ion.ucl.ac.uk/spm/

4
https://de.mathworks.com
