# Supplementary material for: Neural classification maps for distinct word combinations in Broca’s area
Source: Front Hum Neurosci. 2022 Nov 3;16:930849. doi: 10.3389/fnhum.2022.930849 (PMC9671167; doi:10.3389/fnhum.2022.930849)
Supplement: Supplementary file 1 [file Data_Sheet_1.PDF]

Supplementary Materials

Neural classification maps for distinct word combinations in Broca's area

Marianne Schell, Angela D. Friederici and Emiliano Zaccarella

Stimuli list

The DP condition consisted of simple determiners with inflectional gender marking, followed by nouns. We follow influential linguistic theories suggesting that articles (e.g., the), demonstratives (e.g., this), quantifiers (e.g., some) and other numerals (e.g., two) belong to the DP system, which may occupy distinct (high, middle, low) structural positions within the DP to determine the interpretation of meaning (Zamparelli, 2004). The purpose of this work is to focus on the first aspect, namely the similarity of function across determiners, to investigate how the combinations between distinct categories (here determiners and adjectives with nouns) may be differentiated at the neural level—regardless of whether different DP combinations (e.g., every book vs. this book) have a theoretically-internally motivated structural complexity that guides the mapping to semantic meaning. A first partial attempt in this respect is found in a very recent behavioural study that uses eye-tracking methodology to recapitulate the predictability of distinct parsing models for the processing of DP/NP phrases formed by different person features (i.e., second person plural pronouns and plural definite determiners introducing nouns) as a function of complexity (Chesi and Canal, 2019). The NP condition consisted of qualifying adjectives for the first phrase with respective inflectional ending and colour adjectives for the second phrase, counterbalanced across grammatical gender. Each participant received a separate subset list of items from the set of all possible DP or NP combinations. Determiners: dies +er (masculine)/+e (feminine)/+es (neutrum), *this*; jen -er/-e/-es, *that*; jed -er/-e/-es, *each*; manch -er/-e/-es, *some*. Adjectives: lang -er/-e/-es, *long*; kurz -er/-e/-es, *short*; groß -er/-e/-es, *big*; klein -er/-e/-es, *small*; rot -er/-e/-es, *red*; gelb -er/-e/-es, *yellow*; blau -er/-e/-es, *blue*; grün -er/-e/-es, *green*. Synonym pairs (light green

background below): Büchse, *can* | Dose, can; Fahne, *flag* | Flagge, *flag*; Flasche, *bottle* | Pulle, *bottle*; Schaufel, *shovel* | Schippe, *shovel*; Besen, *broom* | Feger, *sweeper*; Hocker, *stool* | Schemel, *stool*; Pfeiler, *pillar* | Pfosten, *post*; Traktor, *tractor* | Trecker, *tractor*; Betttuch, *sheet* | Laken, *sheet*; Bildnis, *portrait* | Portrait, *portrait*; Plakat, *poster* | Poster, *poster*; Symbol, *symbol* | Zeichen, *sign.* Additional nouns: Kerze, *candle*; Kette, *chain*; Spritze, *syringe*; Trage, *stretcher*; Kragen, *collar*; Pfropfen, *plug*; Stecker, *jack*; Tresor, *safe*; Messer, *knife*; Puzzle, *puzzle*; Segel, *sail*; Zepter, *sceptre*.

Possible DP combinations

diese	Buechse	jede	Buechse	jene	Buechse	manche	Buechse
diese	Dose	jede	Dose	jene	Dose	manche	Dose
diese	Fahne	jede	Fahne	jene	Fahne	manche	Fahne
diese	Flagge	jede	Flagge	jene	Flagge	manche	Flagge
diese	Flasche	jede	Flasche	jene	Flasche	manche	Flasche
diese	Kerze	jede	Kerze	jene	Kerze	manche	Kerze
diese	Kette	jede	Kette	jene	Kette	manche	Kette
diese	Pulle	jede	Pulle	jene	Pulle	manche	Pulle
diese	Schaufel	jede	Schaufel	jene	Schaufel	manche	Schaufel
diese	Schippe	jede	Schippe	jene	Schippe	manche	Schippe
diese	Spritze	jede	Spritze	jene	Spritze	manche	Spritze
diese	Trage	jede	Trage	jene	Trage	manche	Trage
dieser	Besen	jeder	Besen	jener	Besen	mancher	Besen
dieser	Feger	jeder	Feger	jener	Feger	mancher	Feger
dieser	Hocker	jeder	Hocker	jener	Hocker	mancher	Hocker
dieser	Kragen	jeder	Kragen	jener	Kragen	mancher	Kragen
dieser	Pfeiler	jeder	Pfeiler	jener	Pfeiler	mancher	Pfeiler
dieser	Pfosten	jeder	Pfosten	jener	Pfosten	mancher	Pfosten
dieser	Pfropfen	jeder	Pfropfen	jener	Pfropfen	mancher	Pfropfen
dieser	Schemel	jeder	Schemel	jener	Schemel	mancher	Schemel
dieser	Stecker	jeder	Stecker	jener	Stecker	mancher	Stecker
dieser	Traktor	jeder	Traktor	jener	Traktor	mancher	Traktor
dieser	Trecker	jeder	Trecker	jener	Trecker	mancher	Trecker
dieser	Tresor	jeder	Tresor	jener	Tresor	mancher	Tresor
dieses	Betttuch	jedes	Betttuch	jenes	Betttuch	manches	Betttuch
dieses	Bildnis	jedes	Bildnis	jenes	Bildnis	manches	Bildnis
dieses	Laken	jedes	Laken	jenes	Laken	manches	Laken
dieses	Messer	jedes	Messer	jenes	Messer	manches	Messer
dieses	Plakat	jedes	Plakat	jenes	Plakat	manches	Plakat
dieses	Portrait	jedes	Portrait	jenes	Portrait	manches	Portrait
dieses	Poster	jedes	Poster	jenes	Poster	manches	Poster
dieses	Puzzle	jedes	Puzzle	jenes	Puzzle	manches	Puzzle
dieses	Segel	jedes	Segel	jenes	Segel	manches	Segel
dieses	Symbol	jedes	Symbol	jenes	Symbol	manches	Symbol
dieses	Zeichen	jedes	Zeichen	jenes	Zeichen	manches	Zeichen
dieses	Zepter	jedes	Zepter	jenes	Zepter	manches	Zepter

Possible NP combinations

blaue	Buechse	gelbe	Buechse	gruene	Buechse	rote	Buechse
blaue	Dose	gelbe	Dose	gruene	Dose	rote	Dose
blaue	Fahne	gelbe	Fahne	gruene	Fahne	rote	Fahne
blaue	Flagge	gelbe	Flagge	gruene	Flagge	rote	Flagge
blaue	Flasche	gelbe	Flasche	gruene	Flasche	rote	Flasche
blaue	Kerze	gelbe	Kerze	gruene	Kerze	rote	Kerze
blaue	Kette	gelbe	Kette	gruene	Kette	rote	Kette
blaue	Pulle	gelbe	Pulle	gruene	Pulle	rote	Pulle
blaue	Schaufel	gelbe	Schaufel	gruene	Schaufel	rote	Schaufel
blaue	Schippe	gelbe	Schippe	gruene	Schippe	rote	Schippe
blaue	Spritze	gelbe	Spritze	gruene	Spritze	rote	Spritze
blaue	Trage	gelbe	Trage	gruene	Trage	rote	Trage
blauer	Besen	gelber	Besen	gruener	Besen	roter	Besen
blauer	Feger	gelber	Feger	gruener	Feger	roter	Feger
blauer	Hocker	gelber	Hocker	gruener	Hocker	roter	Hocker
blauer	Kragen	gelber	Kragen	gruener	Kragen	roter	Kragen
blauer	Pfeiler	gelber	Pfeiler	gruener	Pfeiler	roter	Pfeiler
blauer	Pfosten	gelber	Pfosten	gruener	Pfosten	roter	Pfosten
blauer	Pfropfen	gelber	Pfropfen	gruener	Pfropfen	roter	Pfropfen
blauer	Schemel	gelber	Schemel	gruener	Schemel	roter	Schemel
blauer	Stecker	gelber	Stecker	gruener	Stecker	roter	Stecker
blauer	Traktor	gelber	Traktor	gruener	Traktor	roter	Traktor
blauer	Trecker	gelber	Trecker	gruener	Trecker	roter	Trecker
blauer	Tresor	gelber	Tresor	gruener	Tresor	roter	Tresor
blaues	Betttuch	gelbes	Betttuch	gruenes	Betttuch	rotes	Betttuch
blaues	Bildnis	gelbes	Bildnis	gruenes	Bildnis	rotes	Bildnis
blaues	Laken	gelbes	Laken	gruenes	Laken	rotes	Laken
blaues	Messer	gelbes	Messer	gruenes	Messer	rotes	Messer
blaues	Plakat	gelbes	Plakat	gruenes	Plakat	rotes	Plakat
blaues	Portrait	gelbes	Portrait	gruenes	Portrait	rotes	Portrait
blaues	Poster	gelbes	Poster	gruenes	Poster	rotes	Poster
blaues	Puzzle	gelbes	Puzzle	gruenes	Puzzle	rotes	Puzzle
blaues	Segel	gelbes	Segel	gruenes	Segel	rotes	Segel
blaues	Symbol	gelbes	Symbol	gruenes	Symbol	rotes	Symbol
blaues	Zeichen	gelbes	Zeichen	gruenes	Zeichen	rotes	Zeichen
blaues	Zepter	gelbes	Zepter	gruenes	Zepter	rotes	Zepter

Phrases in first position

grosse	Dose	kurze	Dose
grosse	Flagge	kurze	Flagge
grosse	Flasche	kurze	Flasche
grosse	Schippe	kurze	Schippe
grosser	Feger	kurzer	Feger
grosser	Pfeiler	kurzer	Pfeiler
grosser	Schemel	kurzer	Schemel
grosser	Traktor	kurzer	Traktor
grosses	Betttuch	kurzes	Betttuch
grosses	Bildnis	kurzes	Bildnis
grosses	Plakat	kurzes	Plakat
grosses	Zeichen	kurzes	Zeichen
kleine	Dose	lange	Dose
kleine	Flagge	lange	Flagge
kleine	Flasche	lange	Flasche
kleine	Schippe	lange	Schippe
kleiner	Feger	langer	Feger
kleiner	Pfeiler	langer	Pfeiler
kleiner	Schemel	langer	Schemel
kleiner	Traktor	langer	Traktor
kleines	Betttuch	langes	Betttuch
kleines	Bildnis	langes	Bildnis
kleines	Plakat	langes	Plakat
kleines	Zeichen	langes	Zeichen

Filler items

blaue	jene
blauer	dieser
blaues	jedes
gelbe	manche
gelber	dieser
gelbes	jedes
gruene	jene
gruener	dieser
gruenes	manches
rote	jede
roter	jener
rotes	manches
diese	jede
dieser	jener
dieses	jenes
jede	diese
jeder	mancher
jedes	manches
jene	jede
jener	jeder
jenes	dieses
manche	diese
mancher	jener
manches	jedes

Bidirectional Rating. We run bidirectional ratings (Whitten, Newton Suter and Frank, 1979) on semantic similarities of German noun pairs to create the experimental stimuli. We first selected all bi-syllabic nouns from the Leipziger Wortschatz database (wortschatz.uni-leipzig.de). All nouns referring to abstract concepts or having any intrinsic color that could not easily be matched with color adjectives were first removed (e. g. animals or fruits). We then selected 106 pairs of synonyms, consisting of 163 different single nouns, since some of the nouns were used more than once in pairings. All synonyms were matched with an unrelated word according to gender. We asked 18 healthy participants (9 female, age: mean=24.7, std =2.8) to rate each word pair for semantic similarities on a computer screen. All pairs were assigned to two runs, each run comprised all 106 synonym pairs (half of the pairs in forward order, half of them in reverse order) and a corresponding unrelated word pair (total number of items = 424). Every trial started with a fixation cross. The first word was the presented on the screen for 2 secs, followed by the second word which stayed on the screen until participants rated their semantic similarity on a 7-point Likert scale, starting from 1 “not at all similar” to 7, “very much similar”. The experiment was spitted into blocks, each with 52 trials, inserted by a 5 min break to reduce fatigue and habituation effects. For each participant and quadruple of words, we received four ratings, two for the synonym pair, for forward and backward rating, and two for synonym and unrelated word pair. We calculated the mean button press across all pairs [synonyms: mean=5,213, SD= 0,903; unrelated: mean=1,431, SD=0,461]; and the grand mean between the forward and the reversed order of each synonym pair. For each pair of synonyms, we run paired t-tests between the forward and the reversed rating scores across all participants, which enables us to predict directionality of synonyms. Negative t-values

indicate a higher rating for the reversed pair, whereas positive t-values indicate a higher rating for the forward pair. This implies that no or small word order effect refer to a t-values around zero. We selected 12 unique pairs of synonyms with the highest ratings of semantic similarity (>6) and no significant effect for directionality for the functional MRI experiment. Additionally, we chose the corresponding unrelated word with the lowest similarity rating.

Fig. S1

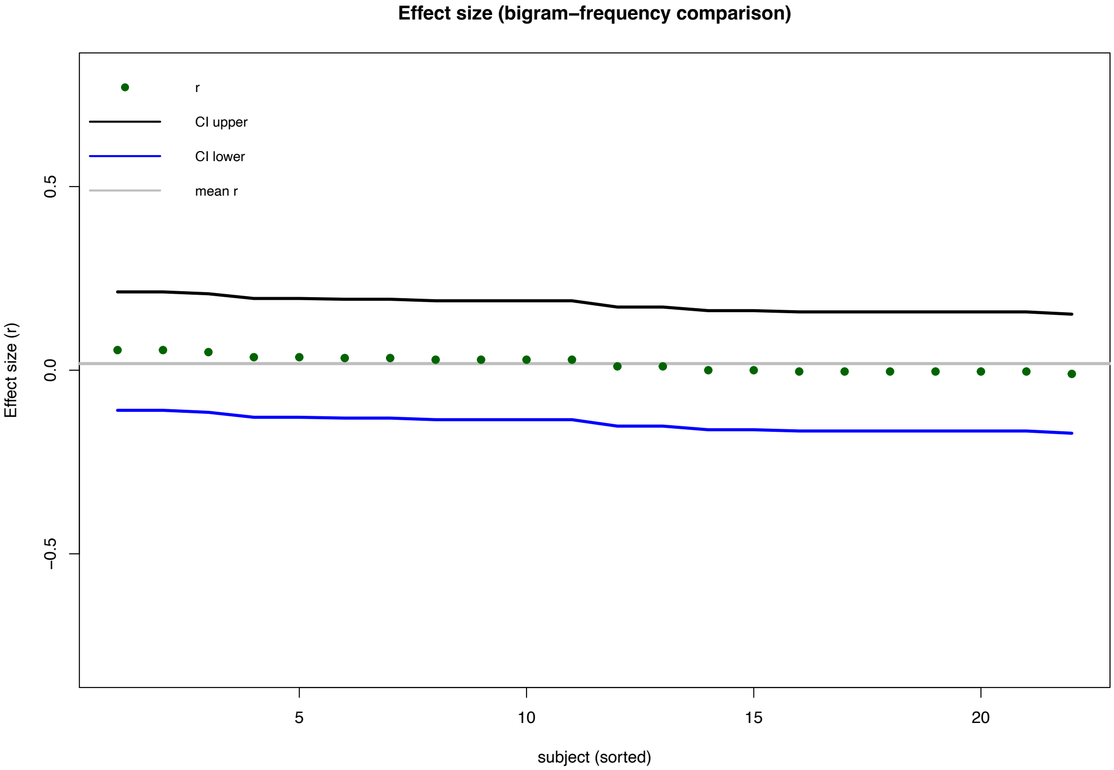

Bigram frequency comparison between DP and NP combinations. For each subject, items were matched for syllabic length and controlled for bigram frequency using the google web1t database containing n-gram counts for approximately 100 billion word-tokens for the German language (Linguistic Data Consortium, University of Pennsylvania). Average bigram frequencies (log) across participants were 2.28 (SD: 0.10) for the DPs and 2.12 (SD: 0.10) for the NPs, with an averaged effect size (r) obtained from a Mann-Whitney's U Test of 0.018 (C.I. =-0.14-0.17). Bigrams with an occurrence value of 0, were assigned a value of 1 before log-transformation, these were 66.5% on average for both conditions. Green dots represent effect size values for each subject, sorted from high-to-low. Black and blue lines are the upper and lower Confidence Intervals (C.I.) respectively. The grey line corresponds to the averaged effect size across subjects. To note, a recent

fMRI study in German language using word frequency as parametric regression in a univariate analysis found that both pars triangularis and pars opercularis of the IFG (i.e., BA45 and BA44, respectively) showed an activation decrease as a function of increasing word frequency, thus suggesting that frequency might not be a source of differentiation within the region (Schuster *et al.*, 2016).

Audio Recordings. All words were individually recorded by a trained male speaker, digitized (16-bit, 44.1 kHz sampling rate, mono) and normalized according to root-mean-square amplitude of all single files. All phrases were created by adding 40msec between the first word and the noun, and by adding 400msec between the two phrases in each trial. Finally, all trials were embedded in speech-shape noise starting 100ms earlier to the trial to prepare the participants for the upcoming trial (Schell, Zaccarella and Friederici, 2017).

Fig. S2

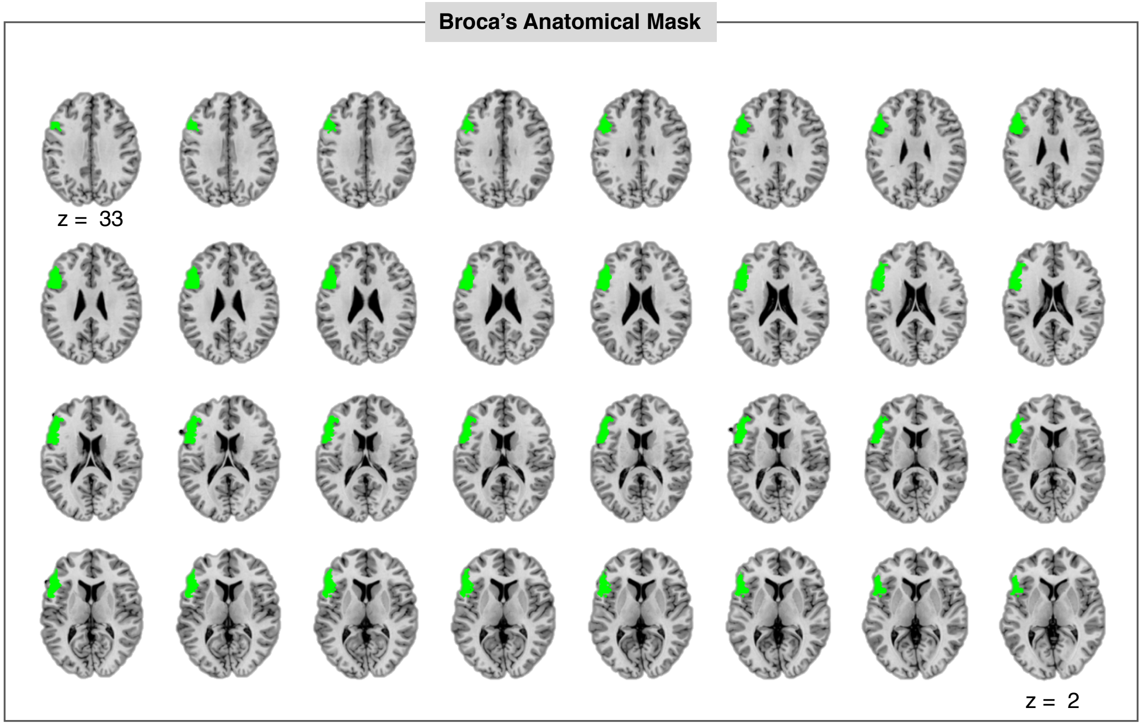

Broca's anatomical mask. Broca's area was defined with cytoarchitectonic maps(Eickhoff *et al.*, 2005) of area 44 and 45 of the left hemisphere. Axial slices from z = 33 to z = 2. Figure created using the Multi-Image Analysis GUI viewer (Mango, Version 4.1; http://ric.uthscsa.edu/mango/).

Fig. S3

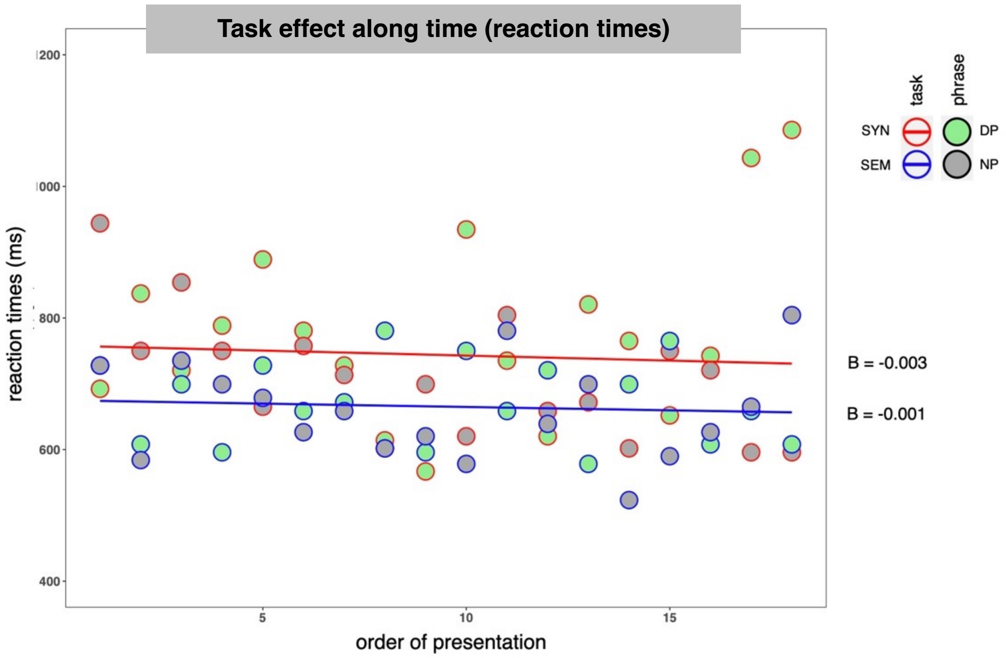

Task effect along time. Relationship between reaction times (RT) for phrase trials and corresponding order of presentation in the study with linear slopes for each task separately (SYN, red line and SEM, blue line). Each dot represents the median RT value response for each phrase trial across subjects—DP_SYN (black dot, red stroke), DP_SEM (black dot, blue stroke), NP_SYN (grey dot, red stroke), NP_SEM (grey dot, blue stroke). The two slopes look extremely parallel, with very marginal negative tendency along the experiment. We tested for differences between the two slopes using linear regression. We failed to reject the hypothesis that a significant interaction in the relationship between RT and order of trial exists, SYN (B = -0.003), SEM (B = -0.001), $F(1, 68) = 0.08$, $p = 0.78$, pointing towards some similar learning trend for both tasks.

Fig. S4

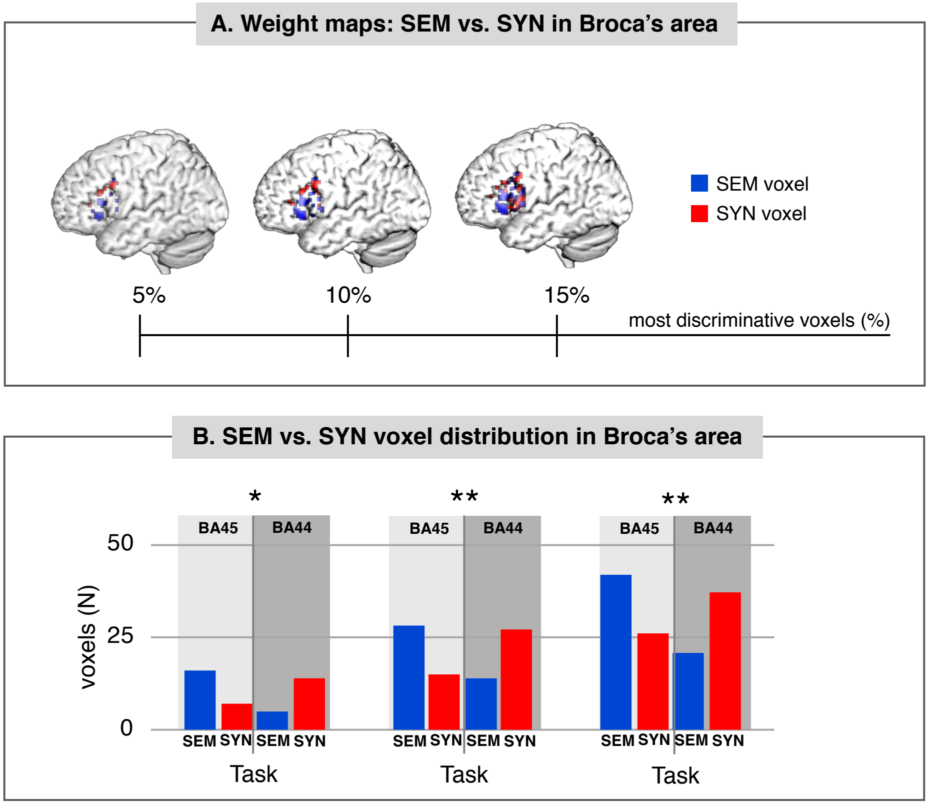

TASK classification patterns. (A) Weight maps: NP vs. DP in Broca's area. The most discriminative 5%, 10% and 15% of classifier weights from the classifier trained to discriminate DP vs. NP, along the PHRASE factor in Broca's area. (B) NP vs. DP voxel distribution in Broca's area. PHRASE classification χ^2-tests showed significantly distinct distributional patterns in Broca's area for 5the % (left), 10% (middle) and 15% (right) most discriminative voxels, with voxels identifying determiner-noun combinations being strongly located in BA 44, while voxels classifying adjective-noun combinations being conversely located in BA 45. * $p < 0.01$, ** $p < 0.005$. Figure created using the Multi-Image Analysis GUI viewer (Mango, Version 4.1; http://ric.uthscsa.edu/mango/) and MATLAB 7.14.0.739 (R2012a, Mathworks, Inc., Sherborn, MA, USA).

Fig. S5

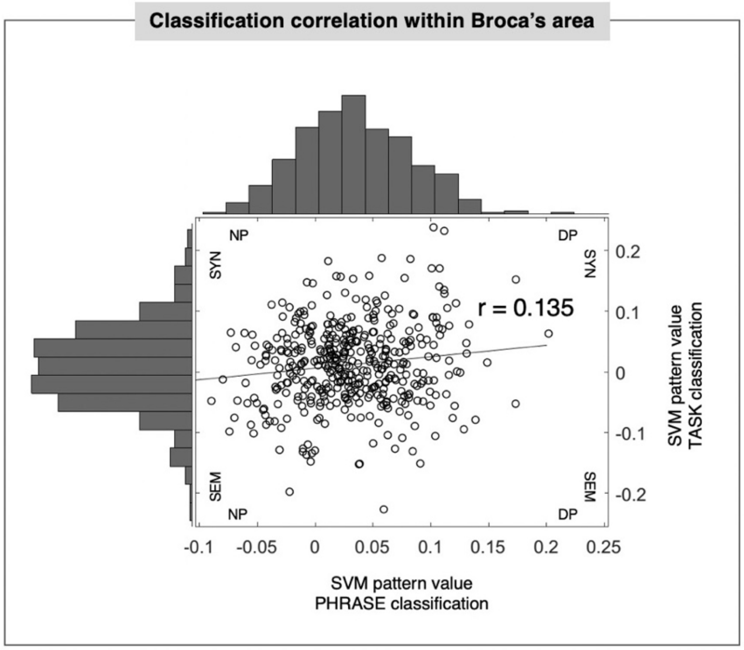

Classification correlation within Broca's area. A linear relationship between PHRASE and TASK was tested to see if those voxels being the most informative for one classification where also the most informative for the other classification. Pearson: r = 0.135; p = 0.005. Figure made with MATLAB 7.14.0.739 (R2012a, Mathworks, Inc., Sherborn, MA, USA).

Fig. S6

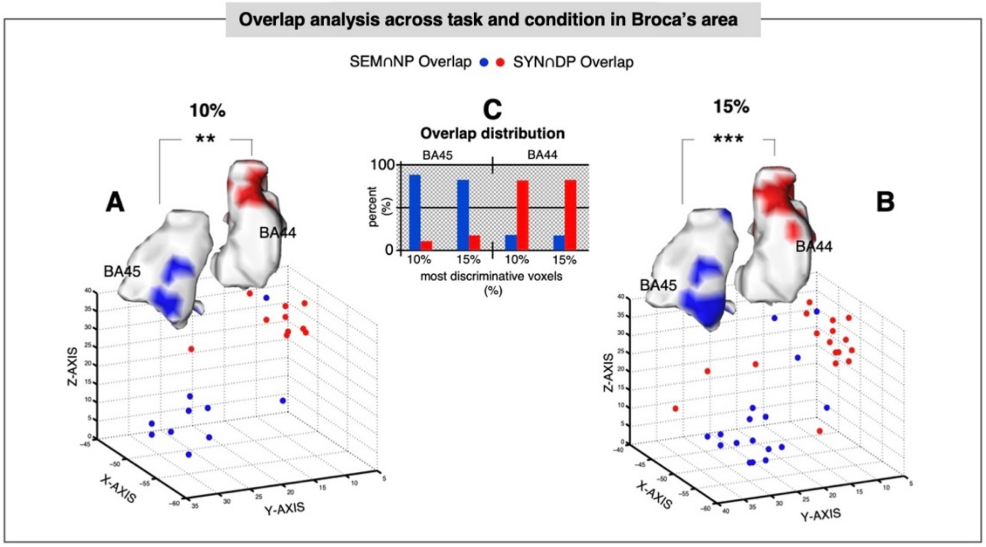

Overlap analysis across task and condition in Broca's area. We performed additional explorative overlap analysis in Broca's area and searched for voxels showing classification for both DP (independent of task) and for syntactic task (independent of condition). Overall, the goal of this post-hoc analysis was to observe whether phrases and tasks best decoded in one region would also show some spatial overlap in that region. Since the overlapping degree at 5% was barely manifest and only ranged between $0 < N_{VOXELS} < 3$ across conditions, we restricted our analysis to the best 10% and 15% of the coding voxels. (A) The 10% analysis yielded 10 unspecific voxels for syntax and 10 for semantics. These voxels showed the following distribution: AREA(TASK∩CONDITION) = N_{VOXELS} (%): $BA45_{(SEM∩NP)}$ = 8 (88.88%); $BA45_{(SYN∩DP)}$ = 1 (11.11%); $BA44_{(SEM∩NP)}$ = 2 (18.18%); $BA44_{(SYN∩DP)}$ = 9 (81.81%). Fisher's Exact Test was significant at $p < 0.01$. (B) The 15% analysis yielded 17 unspecific voxels for syntax and 17 for semantics with the following distributions: $BA45_{(SEM∩NP)}$ = 14 (82.35%); $BA45_{(SYN∩DP)}$ = 3 (17.64%); $BA44_{(SEM∩NP)}$ =

3 (17.64%); $BA44_{(SYN \cap DP)}$ = 14 (82,35%). Fisher's Exact Test was significant at $p < 0.001$. (C) Overlap distribution showing voxel overlap for BA44 and BA45. ** $p = 0.01$, *** $p < 0.001$ (Fisher's Exact Test). Figure created using the Multi-Image Analysis GUI viewer (Mango, Version 4.1; http://ric.uthscsa.edu/mango/) and MATLAB 7.14.0.739 (R2012a, Mathworks, Inc., Sherborn, MA, USA).

Fig. S7

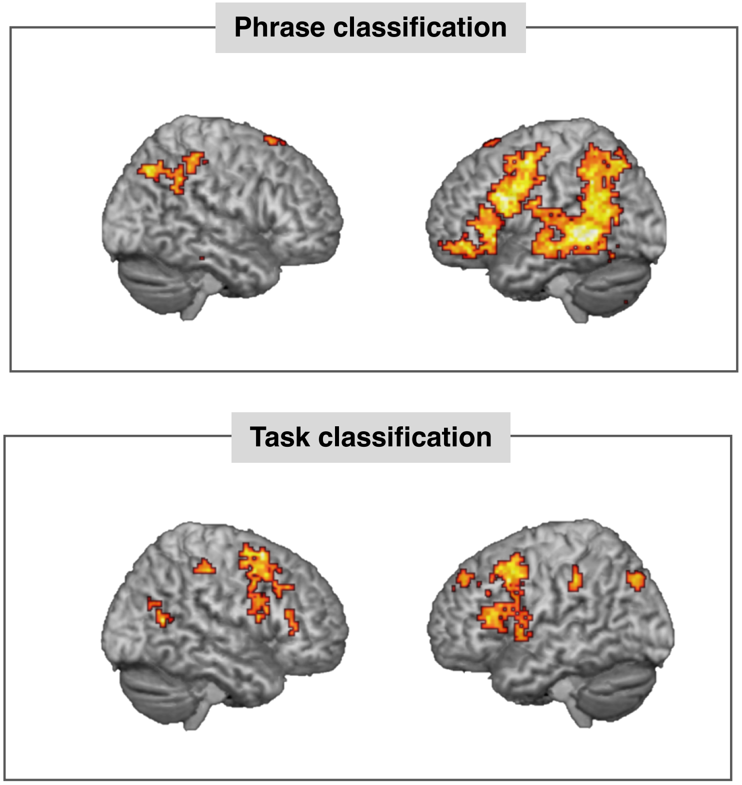

Whole-brain searchlight analysis. The MVPA was performed on a leave-one-run-out cross-validation with six folds. For every voxel a classifier was trained using all voxels within a spherical searchlight with a radius 12mm (equals 4 voxels). This was performed iteratively for all voxels in the brain, which yields to a 3-dimensional accuracy map, where each voxel value derived from the information present in its searchlight, not the voxel individually. The mean accuracy was subtracted from 50% (chance accuracy level for a two-class decision) and assigned to the voxel in the center of the sphere across all cross-validation steps. This was repeated iteratively for all voxels in the brain, resulting in an

individual three-dimensional image with accuracy over chance values for every voxel in the brain. Group analysis was performed by combining the individual subject maps with a t-test at each voxel as implemented in SPM8 to evaluate the classification approach ($p < 0.001$ uncorrected, $p < 0.05$ FWE cluster-corrected). Figure created using the Multi-Image Analysis GUI viewer (Mango, Version 4.1; http://ric.uthscsa.edu/mango/).

References

Chesi, C. and Canal, P. (2019) 'Person Features and Lexical Restrictions in Italian Clefts', *Frontiers in Psychology*, 10. doi: 10.3389/fpsyg.2019.02105.

Eickhoff, S. B. *et al.* (2005) 'A new SPM toolbox for combining probabilistic cytoarchitectonic maps and functional imaging data', *Neuroimage*. 2005/04/27, 25(4), pp. 1325–1335. doi: 10.1016/j.neuroimage.2004.12.034.

Schell, M., Zaccarella, E. and Friederici, A. D. (2017) 'Differential cortical contribution of syntax and semantics: An fMRI study on two-word phrasal processing', *Cortex*, 96, pp. 105–120. doi: 10.1016/j.cortex.2017.09.002.

Schuster, S. *et al.* (2016) 'Words in Context: The Effects of Length, Frequency, and Predictability on Brain Responses during Natural Reading', *Cerebral Cortex*. Oxford University Press, 26(10), pp. 3889–3904. doi: 10.1093/cercor/bhw184.

Whitten, W. B., Newton Suter, W. and Frank, M. L. (1979) 'Bidirectional synonym ratings of 464 noun pairs', *Journal of Verbal Learning and Verbal Behavior*. Academic Press, 18(1), pp. 109–127. doi: 10.1016/S0022-5371(79)90604-2.

Zamparelli, R. (2014) *Layers in the Determiner Phrase*. Routledge. doi: 10.4324/9781315054360.
